# Bochdalek Hernia and Partial Diaphragmatic Agenesis: Pedicled Intercostal Muscle Flap and Mesh Repair in a Young Adult with Sickle Cell Disease

**DOI:** 10.1055/s-0041-1740628

**Published:** 2021-12-23

**Authors:** Klein Dantis, Devendra Kumar Rathore, Nilesh Gupta, Subrata Kumar Singha

**Affiliations:** 1 Department of Cardiothoracic Surgery, All India Institute of Medical Sciences, Raipur, Chhattisgarh, India; 2 Department of General Surgery, All India Institute of Medical Sciences, Raipur, Chhattisgarh, India; 3 Department of Radiodiagnosis, All India Institute of Medical Sciences, Raipur, Chhattisgarh, India; 4 Department of Anesthesiology, All India Institute of Medical Sciences, Raipur, Chhattisgarh, India

**Keywords:** congenital diaphragmatic hernia, hemidiaphragm agenesis, sickle cell disease, intercostal muscle flap, ectopic kidney, vertebral fusion

## Abstract

Congenital Bochdalek hernia (BH) in an adult is rare and has an unusual presentation. They are confined to the pediatric age group with an incidence of 1:3,000 live births. It rarely persists asymptomatic until adulthood. Surgical repair by thoracic, abdominal, or thoraco-abdominal approach is the treatment of choice with diaphragmatic reconstruction in associated diaphragmatic agenesis. With only 10 cases of BH with partial diaphragmatic agenesis reported to date, we discuss the rarity, unusual presentation, and management of BH in a young adult with sickle cell disease that has not been reported in the literature.

Adult congenital Bochdalek hernia (BH) is uncommon and usually confined to the pediatric population with an incidence of 1:3,000 live births, 0.17% on abdominal computed tomography, 1 and prevalence of 1 in 2,000–7,000 autopsy study. 1 2 Etiology is explained by the incomplete fusion of pleuroperitoneal folds during the 9th–10th weeks of pregnancy, leading to the persistent pleuroperitoneal canal. Though the left side predominates, the right side is frequently seen in adults and detected incidentally (5%) during other investigations. 3 In a young adult with sickle cell disease, we now discuss the rarity and management of BH, associated ectopic kidney, and vertebral fusion with partial diaphragmatic agenesis.

## Case Report

**Fig. 1:**
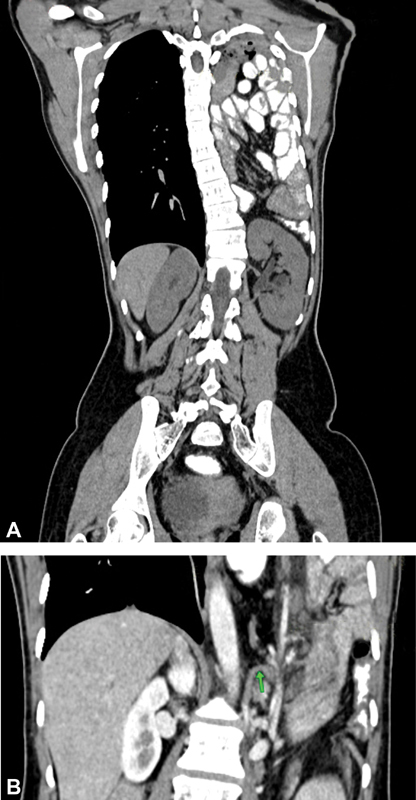
( **A** ) Contrast-enhanced computed tomography (CECT) of the thorax and abdomen showing bowel loops in the left thoracic cavity with ectopic kidney and thoracolumbar scoliosis. ( **B** ) CECT thorax showing displaced left diaphragmatic crus (as indicated by an arrow in green) with significant defect.

A 24-year-old female with a sickle cell disease presented with mild chest pain, an episode of vomiting, and no history of trauma or chronic illness; she was hemodynamically stable, afebrile with regular abdomen examination. Chest examination revealed decreased left air entry, chest X-ray showed left consolidation, and ultrasound thorax confirmed the presence of bowel loops and left kidney within the thoracic cavity. Contrast-enhanced computed tomography (CECT) of the thorax and abdomen showed colon, small bowel, left kidney in the thoracic cavity with scoliosis (Cobb angle of 24 degrees), fused 12th thoracic and 1st lumbar vertebral bodies, and displaced left diaphragmatic crus and its defect ( Fig. 1A , 1B ).

**Fig. 2:**
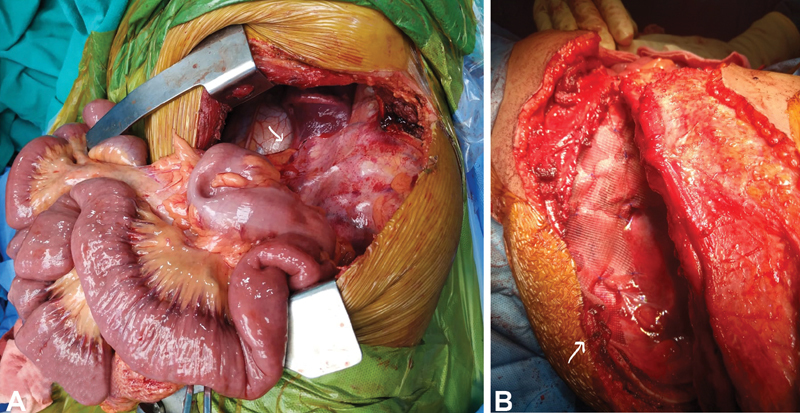
( **A** ) Intraoperative finding: mesentery of bowel loops adherent to the adventitia of aorta and pericardium with no bowel ischemia. ( **B** ) Diaphragmatic reconstruction: polypropylene mesh placed over primary repair and intercostal flap rotated toward diaphragmatic floor.

**Fig. 3:**
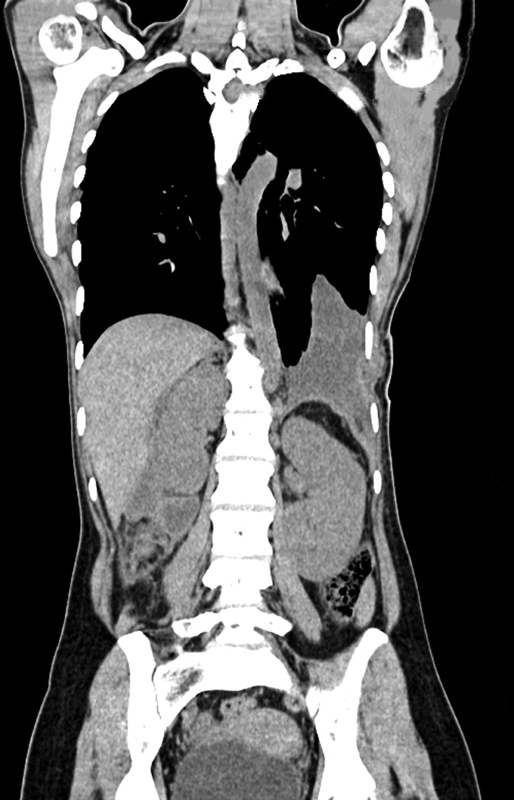
Postoperative contrast-enhanced computed tomography showing neo-diaphragm with minimal pleural effusion and no recurrence at 14 days.

The patient received preoperative antibiotics, nebulization, and bowel preparation with normal blood investigation. Following left thoracotomy, the thoracic cavity was accessed through the fifth intercostal space, which contained a hypoplastic lung, the colon, small intestine up to the duodenal jejunal flexure, and left kidney with no hernia sac. The normal colonic mesentery was adherent to the aorta and pericardium ( Fig. 2A ). Following vascular adhesiolysis and laparotomy, intrathoracic contents were transpositioned to normal anatomical positions. Primary partial diaphragmatic repair, pedicled intercostal muscle flap raised between the sixth and seventh ribs, followed by partial excision of the sixth rib obliterated posterolateral defect. Polypropylene mesh was placed over the primary repair and around the flap with Prolene 3-0 to provide diaphragmatic stability ( Fig. 2B ). A single drain was placed in the chest and abdomen, followed by a closure. Mild splenomegaly was not an indication for splenectomy. Extubated patient was maintained nil by mouth for 24 hours. She received one unit (350 mL) of packed red blood cells and Ryles tube feeding with fluids on the first and second postoperative days followed by oral feeds and a soft diet from the third and fourth days. The postoperative course was uneventful, with chest and abdomen drains removed on the eighth and ninth days. CECT thorax and abdomen repeated after 2 weeks revealed minimal pleural effusion with an intact diaphragm that resolved with subsequent follow-up visits and no recurrence ( Fig. 3 ) with CECT obtained at the third and sixth months.

## Discussion

Czech anatomist Vincent Alexander Bochdalek first described congenital adult diaphragmatic hernia in 1848. 1 Due to varied clinical presentations, diagnosis is late. 4 Volvulus, strangulation, organ perforation, and tension pneumothorax require emergency intervention. 4 Contents include omentum (92%), splenic flexure of the colon (58%), stomach (25%), spleen and liver. 3

Displaced diaphragmatic crus is explained by the mal-development of at least three of the four parts of the hemidiaphragm anlage, the unpaired ventral portion from the septum transversum, the unpaired dorsolateral portions from the pleuroperitoneal membranes, or the medial portion from persisting parts of the primary mesentery. 5 Associated ectopic intrathoracic kidney accounts for 5% with a prevalence of < 0.01% and the presence of vertebral fusion has been explained as a complex synchronous metameric defect of somite and intermediate mesoderm. 1 6

Surgical repair is the treatment of choice, either by thoracoscopic/laparoscopic approach or by an open repair. We performed an open thoraco-abdominal approach as repositioning of intrathoracic content into the abdominal cavity was not possible through the defect as mesentery of the colon was adherent to the descending thoracic aorta and pericardium as well as dense adhesions within the defect and adhesions confined to the abdominal wall restricted manual reduction. Acute splenic sequestration crises are a complication of sickle cell disease. However, as there is a lack of evidence from the randomized control trials showing that splenectomy improves survival and decreases morbidity in patients with sickle cell disease, hence splenectomy was not considered. 7

Our case is unique in presentation. First, its association with intrathoracic ectopic kidney has been reported in less than 0.25% of the cases and further association of intrathoracic ectopic kidney with vertebral fusion has been reported only once in the literature. 6 8 Second, with only 10 cases of partial diaphragmatic agenesis in adults reported to date, this case adds associated rarity in the form of asymptomatic presentation, absent sac with the adhesion of mesentery to the thoracic aorta, pericardium, and reconstruction with pedicled intercostal muscle flap in the patient with sickle cell disease, which has not been reported in the literature.

## Conclusion

Adult congenital BH is rare, can have unusual presentation, and perioperative management in sickle cell disease is challenging, requiring multimodal management.

## References

[1] Schumacher L Gilbert S Congenital diaphragmatic hernia in the adult Thorac Surg Clin 2009 19 04 469 472 20112629 10.1016/j.thorsurg.2009.08.004

[2] Salaçin S Alper B Cekin N Gülmen M K Bochdalek hernia in adulthood: a review and an autopsy case report J Forensic Sci 1994 39 04 1112 1116 8064271

[3] Kumar N Gupta A Rajput D Bochdalek hernia in an adult female with intrathoracic left kidney and splenic flexure of the colon: a rare case report with literature review Pol Przegl Chir 2019 92 02 60 63 32312917 10.5604/01.3001.0013.2439

[4] Akita M Yamasaki N Miyake T Bochdalek hernia in an adult: two case reports and a review of perioperative cardiopulmonary complications Surg Case Rep 2020 6 01 72 10.1186/s40792-020-00833-w 32303918 PMC7165220

[5] Pattern M B HumuTI Embryology 2nd ed. New York McGraw-Hill 1953

[6] Angulo J C Lopez J I Vilanova J R Flores N Intrathoracic kidney and vertebral fusion: a model of combined misdevelopment J Urol 1992 147 05 1351 1353 1569682 10.1016/s0022-5347(17)37562-6

[7] Owusu-Ofori S Remmington T Splenectomy versus conservative management for acute sequestration crises in people with sickle cell disease Cochrane Database Syst Rev 2017 11 11 CD003425 10.1002/14651858.CD003425 29112240 PMC6486322

[8] Sarac M Bakal U Tartar T Canpolat S Kara A Kazez A Bochdalek hernia and intrathoracic ectopic kidney: presentation of two case reports and review of the literature Niger J Clin Pract 2018 21 05 681 686 29735873 10.4103/njcp.njcp_217_17

